# Overestimation of Amphotericin B Resistance in Candida auris with Sensititre YeastOne Antifungal Susceptibility Testing: a Need for Adjustment for Correct Interpretation

**DOI:** 10.1128/spectrum.04431-22

**Published:** 2023-04-10

**Authors:** Maria Siopi, Ilektra Peroukidou, Maria-Ioanna Beredaki, Bram Spruijtenburg, Theun de Groot, Jacques F. Meis, Georgia Vrioni, Athanasios Tsakris, Spyros Pournaras, Joseph Meletiadis

**Affiliations:** a Clinical Microbiology Laboratory, “Attikon” University General Hospital, Medical School, National and Kapodistrian University of Athens, Athens, Greece; b Department of Medical Microbiology and Infectious Diseases, Canisius-Wilhelmina Hospital, Nijmegen, The Netherlands; c Centre of Expertise in Mycology, Radboud University Medical Center/Canisius Wilhelmina Hospital, Nijmegen, The Netherlands; d Department of Microbiology, Medical School, National and Kapodistrian University of Athens, Athens, Greece; Institut Pasteur

**Keywords:** *Candida auris*, amphotericin B, Sensititre YeastOne, wild type upper limit value, antifungal susceptibility testing, resistance, epidemiological cutoff value

## Abstract

Significant variation in minimal inhibitory concentrations (MIC) has been reported for amphotericin B (AMB) and C. auris, depending on the antifungal susceptibility testing (AFST) method. Although the Sensititre YeastOne (SYO) is widely used in routine laboratory testing, data regarding its performance for the AFST of C. auris are scarce. We tested AMB against 65 C. auris clinical isolates with the SYO and the reference methodology by the Clinical and Laboratory Standards Institute (CLSI). The essential agreement (EA, ±1 dilution) between the two methods and the categorical agreement (CA) based on the Centers for Disease Control and Prevention (CDC)’s tentative breakpoint of MIC ≥ 2 mg/L were determined. The SYO wild type upper limit value (WT-UL) was determined using the ECOFFinder. The modal (range) CLSI growth inhibitory MIC was lower than the SYO colorimetric MIC [1(0.25-1) versus 2(1-8) mg/L, respectively]). The CLSI-colorimetric SYO EA was 29% and the CA was 11% (89% major errors; MaE). MaE were reduced when the SYO WT-UL of 8 mg/L was used (0% MaE). Alternatively, the use of SYO growth inhibition endpoints of MIC-1 (75% growth inhibition) or MIC-2 (50% growth inhibition) resulted in 88% CA with 12% MaE and 97% CA with 3% MaE, respectively. In conclusion, SYO overestimated AMB resistance in C. auris isolates when colorimetric MICs, as per SYO instructions and the CDC breakpoint of 2 mg/L, were used. This can be improved either by using the method-specific WT-UL MIC of 8 mg/L for colorimetric MICs or by determining growth inhibition MIC endpoints, regardless of the color.

**IMPORTANCE** Candida auris is an emerging and frequently multidrug-resistant fungal pathogen that accounts for life-threatening invasive infections and nosocomial outbreaks worldwide. Reliable AF is important for the choice of the optimal treatment. Commercial methods are frequently used without prior vigorous assessment. Resistance to AMB was over-reported with the commercial colorimetric method Sensititre YeastOne (SYO). SYO produced MICs that were 1 to 2 twofold dilutions higher than those of the reference CLSI method, resulting in 89% MaE. MaE were reduced using a SYO-specific colorimetric wild type upper limit MIC value of 8 mg/L (0%) or a 50% growth inhibition endpoint (3%).

## INTRODUCTION

Candida auris is an emerging and frequently multidrug-resistant fungal pathogen that accounts for life-threatening invasive infections and nosocomial outbreaks worldwide. Given its high prevalence and regional pattern of resistance, antifungal susceptibility testing (AFST) is crucial in the guidance of therapeutic decisions (1). Moreover, the fact that C. auris acquired resistance to different classes of antifungals warrants constant attention and further underscores the importance of accurate AFST (2–4). While the reference broth microdilution (BMD) methods of CLSI and European Committee for Antimicrobial Susceptibility Testing (EUCAST) are considered to be the gold standard for AFST, their implementation in laboratory routines is hampered by their labor-intensive and time-consuming nature. Sensititre YeastOne (SYO) is a widely used commercial colorimetric assay for the AFST of yeasts, owing to its ready-to-use nature and its ease of use (5). However, SYO was optimized for common *Candida* species, meaning that the testing of new species may be a challenge due to the unique characteristics of each species (growth rate, growth pattern, metabolic status). Given the differences in the MIC distributions generated using SYO and CLSI, SYO-specific epidemiological cutoff (ECOFF) values have been determined for the detection of non-wild type (WT) isolates for several species (6–8). Systematic assessments of SYO performance for C. auris AFST have not been performed (9). To date, the CDC has proposed tentative fluconazole, amphotericin B (AMB), and echinocandin MIC breakpoints for C. auris (10), and species-specific tentative CLSI and EUCAST ECOFF values have also been proposed (11). Still, no SYO ECOFF is available for C. auris versus any antifungal agent.

Currently, echinocandins are indicated as the first-line therapy for candidemia (12, 13). However, in the context of the emergence of breakthrough C. auris infections as well as the therapeutic failures observed among patients treated with echinocandins (3, 4, 14, 15) in conjunction with the high (91%) resistance rate of C. auris to fluconazole (16), treatment with liposomal AMB could be considered as an alternative therapeutic option (17). A recent meta-analysis revealed a relatively low AMB resistance rate of 12% among C. auris isolates (16), whereas resistance up to 27% and 50% was observed with the SYO assay (18) and the Etest strips (19), respectively. Despite the elevated MICs (>1 mg/L), resistance could not be confirmed molecularly, as no mutations within the *ERG3*, *ERG5*, *ERG6*, and/or *ERG10* genes that have been associated with resistance to AMB in *Candida* species have been detected (20, 21). Of note, the CLSI has recently suggested that AMB susceptibility results should be interpreted with caution because of the significant variability in the MIC values for C. auris and AMB across different testing methodologies (22).

Therefore, we evaluated the SYO performance for the AFST of AMB against C. auris, compared to the reference CLSI BMD method in an attempt to compare the two methods and optimize SYO AFST.

## RESULTS

### Colorimetric endpoint.

**FIG 1:**
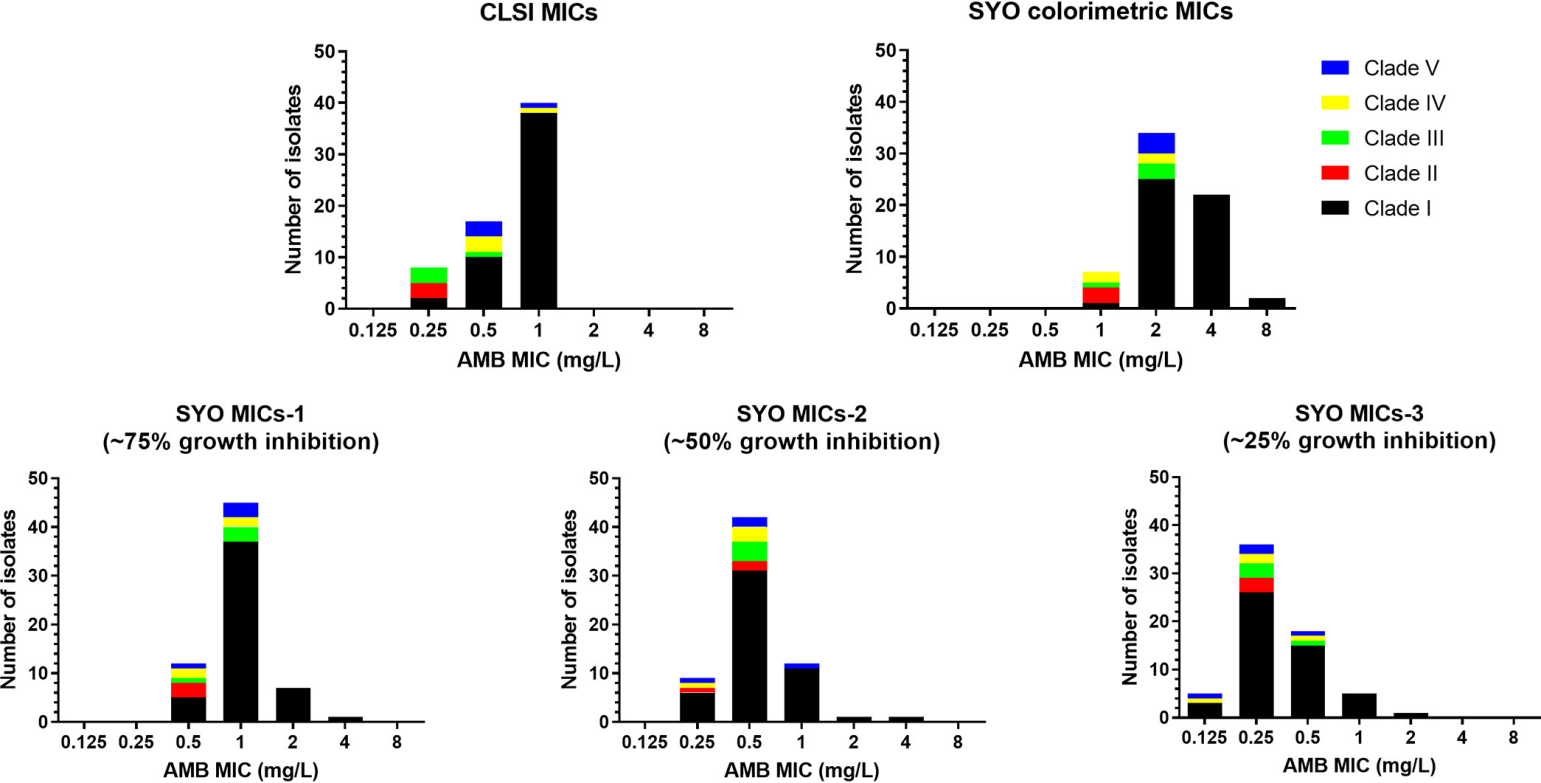
CLSI and Sensititre YeastOne (SYO) MIC distributions of 65 C. auris isolates by clade.

**FIG 2:**
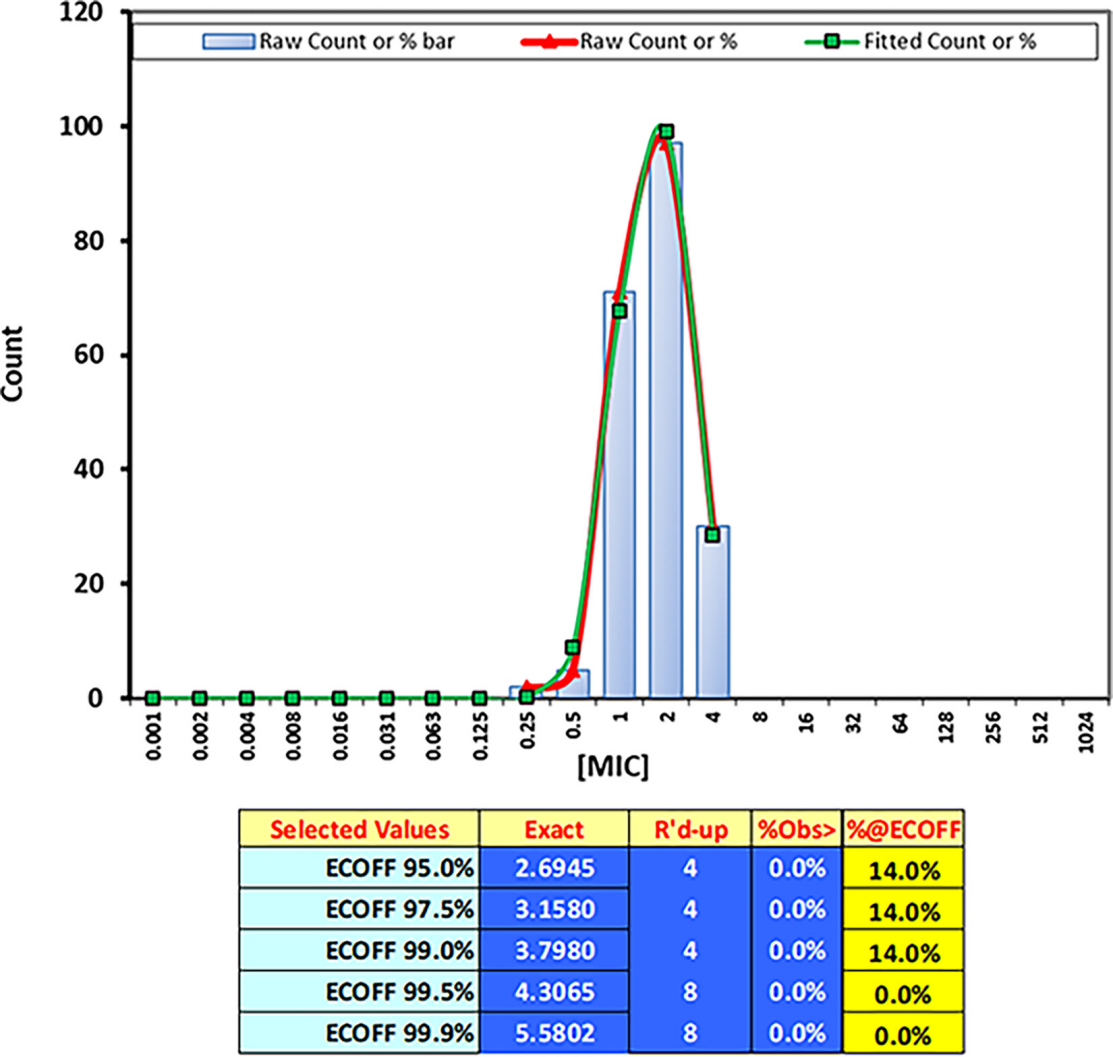
Distribution of colorimetric MICs (first blue well) of 205 C. auris clinical isolates from the ongoing outbreak in Greece, obtained using the Sensititre YeastOne method and analyzed using the ECOFFinder program (31).

Regarding the SYO method, trailing endpoints were not encountered, and the first blue well corresponded to MIC-0 for all isolates. The modal (range) SYO color imetric MIC, MIC_50_, and MIC_90_ values were 2 (1 to 8 mg/L), 2 mg/L, and 4 mg/L, respectively (Table 1). On the other hand, the modal (range) CLSI MIC, MIC_50_, and MIC_90_ values were 1 (0.25 to 1) mg/L, 1 mg/L, and 1 mg/L, respectively (Table 1). The SYO color and CLSI MIC distributions of C. auris isolates by clade are shown in Fig. 1. The interobserver agreement was excellent (100%) for both methods, whereas the MIC values for the quality control strains were within the reference ranges. The absolute interexperimental agreement between replicates of the SYO color endpoint was 70%, whereas the EA (±1 log_2_ dilution) was 100%. Overall, the CLSI-colorimetric SYO EA within ±1 twofold dilution was poor (29%), with a median (range) difference of 2 (0 to 3) twofold dilutions. Greater EA was found within ±2 twofold dilutions (88%). The MIC values that were obtained by the two methods were significantly different (*P* < 0.0001), although they showed a moderate but significant correlation (Spearman’s *r_s_* [95% CI] of 0.53 [0.32 to 0.69], *P* < 0.0001). All isolates were interpreted as susceptible, based on the CLSI MICs, whereas 58 out of 65 strains were resistant to AMB, according to the SYO colorimetric MICs, which corresponded to 11% CA and 89% MaE. The estimated SYO colorimetric WT-UL encompassing >99% of the isolates was 8 mg/L (Table 1). The WT-UL of 8 mg/L was verified in a larger collection of 205 clade I C. auris clinical isolates for the ongoing outbreak in Greece (Fig. 2). Based on a WT-UL of 8 mg/L, all isolates were deemed as susceptible with 100% CA with CLSI.

**TABLE 1:** *In vitro* susceptibility profile of 65 C. auris isolates belonging to different clades, determined using the CLSI and Sensititre YeastOne (SYO) methods^*a*^

Method	Endpoint	No. of isolates with MIC (mg/L) of:							MIC_50_	MIC_90_	% resistance
		0.125	0.25	0.5	1	2	4	8			
CLSI	100% growth inhibition		8	17	40^*b*^				1	1	0
SYO	First blue well				7	34^*b*^	22	2	2	4	89
	Approximately 75% growth inhibition (MIC-1)			12	45^*b*^	7	1		1	2	12
	Approximately 50% growth inhibition (MIC-2)		9	42^*b*^	12	1	1		0.5	1	3
	Approximately 25% growth inhibition (MIC-3)	5	36^*b*^	18	5	1			0.25	0.5	2

a Resistant isolates, based on the CDC’s amphotericin B tentative MIC breakpoint for C. auris, are shaded (10).

b Modal MICs.

### Growth inhibition endpoints.

When the SYO growth inhibition endpoints were taken into account, the modal (range) SYO MIC, MIC_50_, and MIC_90_ values were 1 (0.5 to 4 mg/L) mg/L, 1 mg/L, and 2 mg/L for MIC-1, 0.5 (0.25 to 4 mg/L) mg/L, 0.5 mg/L, and 1 mg/L for MIC-2, and 0.25 (0.125 to 2 mg/L) mg/L, 0.25 mg/L, and 0.5 mg/L for MIC-3, respectively. The MIC data of the C. auris isolates by clade that were obtained using different SYO growth inhibition endpoints are depicted in Fig. 1. The EA values between the CLSI MIC and the SYO MIC-1, MIC-2, and MIC-3 within ±1 twofold dilution were 92%, 90%, and 62%, respectively, with a median (range) difference of 0 (−1 to 2), 0 (−2 to 2), and −1 (−3 to 1) twofold dilutions, respectively. The CLSI-SYO EA values within ±2 twofold dilutions were 100%, 100%, and 97% for SYO MIC-1, MIC-2, and MIC-3, respectively. The MIC values generated by the two methods were significantly different (*P* < 0.0001 to 0.009), although a moderate but significant correlation (Spearman’s *r_s_* [95% CI] 0.44 [0.21 to 0.63], *P* = 0.0003) was only found between the CLSI MIC and the SYO MIC-1. Fewer discrepancies in the interpretation of the susceptibility data were observed, and the CA increased to 88% (8 out of 65 isolates; 12% MaE) for SYO MIC-1, 97% (2 out of 65 isolates; 3% MaE) for SYO MIC-2, and 98% (1 out of 65 isolates; 2% MaE) for SYO MIC-3, respectively (Table 1).

## DISCUSSION

Accurate AMB AFST against C. auris has recently been caught in the spotlight of attention. Herein, a comparative evaluation of SYO and the CLSI reference method showed poor correlation. The SYO color imetric endpoints were 1 to 2 twofold dilutions higher (29% EA) than the CLSI MICs, with the modal CLSI MIC of 1 mg/L being just below the CDC’s breakpoint of 2 mg/L, thereby resulting in considerable interpretation errors (11% CA). Discrepancies were limited when the SYO-specific colorimetric AMB WT-UL of 8 mg/L was used to detect non-WT/resistant isolates (100% CA) and when a 50% growth inhibition endpoint was used, regardless of the color in the well (90% EA, 97% CA).

Currently, CLSI clinical breakpoints for *Candida* species and AMB are not available. Considering that the AMB CLSI MICs are tightly clustered between 0.25 and 1 mg/L, the MIC value of ≥2 mg/L is used as the resistant breakpoint for all *Candida* species, despite the paucity of data to support it (23). In fact, there has been little problem with this cutoff so far, as the vast majority of *Candida* species display MIC values of <1 mg/L. Nevertheless, this is not the case for C. auris, as AMB resistance (CLSI MICs of >1 mg/L) typically ranges up to 30% (24, 25). Of note, the tentative AMB MIC breakpoint that has been proposed by the CDC is only based on a single PK/PD study in a murine model (10). Thus, the use of the CLSI breakpoint as a reliable gold standard for C. auris raises concerns. Meanwhile, the remarkable variability in the AMB MIC distributions of C. auris across different test methods poses an additional challenge (22), thereby calling for the definition of method-specific breakpoints.

To date, studies on C. auris antifungal susceptibility profiles have mainly been conducted by testing a few isolates with MICs that have been determined using the reference methodology. While SYO is widely used in routine clinical laboratories, data on its performance for the AFST of C. auris are limited, and comparative evaluations with the CLSI/EUCAST method are scarce, thereby precluding comparisons. Only Ruiz-Gaitán et al. compared the AMB MICs of 56 Spanish C. auris strains obtained via the SYO and the EUCAST BMD methods, reporting 44.9% and 93.9% EA within ±1 and ±2 twofold dilutions, respectively, which is in line with our findings (29% and 88%, respectively). The SYO AMB MICs were 1 to 2 twofold dilutions higher than were the EUCAST MICs (MIC_50_ value of 0.125 for EUCAST versus 0.5 mg/L for SYO), as was found in the present study for CLSI, but at higher MICs (9). However, an excellent (100%) CA was observed because all of the isolates were susceptible with both methodologies, as opposed to our results (11% CA) (9). Our CLSI AMB susceptibility data are consistent with those of previous reports (modal MIC, MIC_50_, and MIC_90_ values of 1 mg/L, 1 mg/L, and 1 mg/L versus 0.5 to 1 mg/L, 0.5 to 1 mg/L, and 1 to 2 mg/L, respectively) (11, 26, 27), with the majority of the MIC values being gathered at one twofold dilution step lower from the proposed tentative breakpoint of 2 mg/L (22). In our study, the SYO AMB MICs were higher by 1 to 2 twofold dilutions (modal MIC, MIC_50_, and MIC_90_ 2 mg/L, 2 mg/L, and 4 mg/L, respectively), and they thus passed the margin of “susceptibility”, thereby leading to substantial categorical disagreement. Similar disagreement has been previously described for a C. auris bloodstream isolate with a SYO MIC of 2 mg/L (resistant) and a CLSI MIC of 1 mg/L (susceptible) (28). Of note, Ruiz-Gaitán et al. provided data for isolates that were clonal in nature, gave rise to a 10-month hospital outbreak, and belonged to clade III of C. auris. Moreover, retrospective SYO data have been obtained for 42 out of 56 isolates (9). On the other hand, we included 20 strains from diverse geographic origins that spanned all five of the genetically distinct clades of C. auris as well as 45 Greek clade I strains that were collected from 10 different centers during a 22-month period so as to minimize the overrepresentation of clonal isolates that could affect the estimation of the SYO colorimetric WT-UL as well as future comprehensive performance comparisons of the commercial assay. In addition, the CLSI MICs were simultaneously tested alongside the SYO MICs on the same C. auris isolates in order to restrict variation due to inoculum sizes and incubation conditions.

Overall, unimodal CLSI AMB MIC distributions have been described implying a low rate of resistance of C. auris isolates (11, 26, 27), as was also shown by a recent meta-analysis of the global epidemiology of this yeast (12% AMB resistance rate) (16). However, when Maphanga et al. assessed the susceptibility of 394 South African C. auris bloodstream isolates using SYO, they reported a resistance rate of 27%, based on the CDC’s tentative breakpoint (modal MIC, MIC_50_, and MIC_90_ values of 1 mg/L, 1 mg/L, and 2 mg/L, respectively) (18). Intriguingly, when the AMB MICs were redetermined using gradient concentration strips (Etest), the resistance rate decreased to 6%, thereby highlighting the method variability with AMB AFST as well as the need for setting method-specific ECOFFs. Notably, the resistance rate would be 2% if the 8 mg/L SYO colorimetric MIC breakpoint that is proposed here was adopted. Given that 8 mg/L is the maximum AMB concentration contained in SYO panels, resistant isolates will be detected based on the color of a single well. Alternatively, the growth inhibition SYO MIC-2 (97% CA) could be used in order to identify resistant isolates. Since the determination of the SYO MIC-2 may be difficult, MIC-1, which is usually one twofold dilution lower than the colorimetric MIC endpoint, could also be used. However, the MaE are 12%, and resistant isolates with a MIC-1 of 2 mg/L or 4 mg/L will need to be verified via a reference method.

ECOFF values can aid in identifying isolates with elevated MICs and/or are likely to possess an acquired resistance mechanism. The estimated SYO color imetric AMB WT-UL is based mainly on MIC data for Greek isolates belonging to clade I. However, the process of establishing formal ECOFF values requires data from multiple laboratories, which are not currently available. Although clade I is the most prevalent and is characterized by a wider geographical distribution and a higher frequency of bloodstream infections, further SYO multicenter evaluations encompassing more unrelated C. auris isolates that belong to all five different phylogenetic clades are warranted, owing to the variable regional patterns of AMB resistance (16). In addition, strains with known resistance mechanisms were not included in the CLSI-SYO comparison. Nevertheless, the first mechanism contributing to clinical AMB resistance in C. auris, which is conferred by mutations in the sterol-methyltransferase gene *ERG6*, has only recently been identified (2). Further studies are needed to define the prevalence of the aforementioned mutations as well as other mutations among phenotypically AMB-resistant clinical isolates that could be used to optimize the current AFST methodologies for the reliable detection of AMB resistance in C. auris.

Our study demonstrated an overestimation of C. auris AMB resistance with SYO, underscoring that the SYO colorimetric AMB MICs need to be interpreted with caution if the CDC’s tentative breakpoint is used to guide therapeutic decisions. Further multicenter studies are needed to verify current findings and to determine SYO-specific ECOFF values. Laboratories should be aware that C. auris isolates with a SYO colorimetric AMB MIC of ≤8 mg/L indicate susceptibility and that a SYO growth inhibition endpoint could be used to detect resistance.

## MATERIALS AND METHODS

### Isolates.

A total of 45 clinical isolates, mainly bloodstream isolates (41 out of 45; 91%), were collected from individual patients who were hospitalized in 10 Greek tertiary care hospitals that are located in the Attica region from November of 2020 to August of 2022. All of the isolates were identified to the species level using MALDI-TOF mass spectrometry (Bruker Daltonics, Bremen, Germany) and clustered in clade I (South Asian) (29). In addition, 20 genetically distinct clinical strains belonging to all 5 C. auris clades and being isolated from various geographical regions were tested. These comprised of: 5 strains from clade I (South Asian; Brazil, Kuwait, Iran, India, Oman), 3 strains from clade II (East Asian; South Korea, Japan), 4 strains from clade III (African; South Africa, Spain), 4 strains from clade IV (South American; Venezuela, Colombia), and 4 strains from clade V (Iranian; Iran) (29).

### Antifungal susceptibility testing.

**FIG 3:**
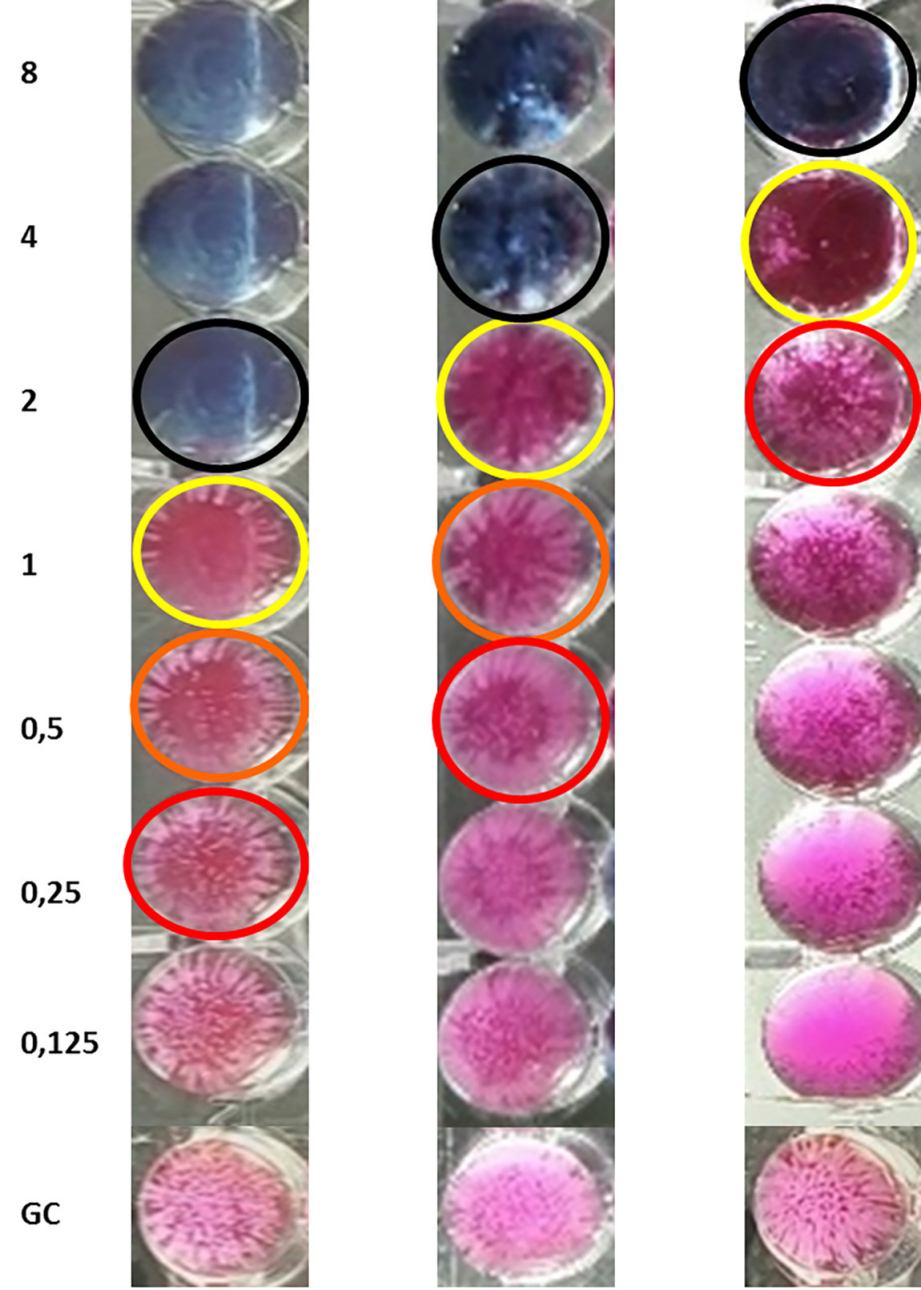
Sensititre YeastOne results for 3 C. auris isolates with colorimetric MICs at (first blue well, black circle) 2 mg/L (first isolate), 4 mg/L (second isolate) and 8 mg/L (third isolate). The growth inhibition endpoints are defined as the lowest drug concentration with no growth (MIC-0, black circle), a substantial reduction of growth (approximately 75% growth inhibition, MIC-1, yellow circle), a prominent reduction of growth (approximately 50% growth inhibition, MIC-2, orange circle), and a slight reduction of growth (approximately 25% growth inhibition, MIC-3, red circle), relative to the drug-free control growth (GC).

The CLSI AFST was performed, according to the M27A4 protocol guidelines, using a laboratory-grade pure AMB powder (Sigma-Aldrich, Athens, Greece). The microtiter plates were incubated at 35 ± 2°C, and the MICs were defined as the lowest drug concentrations at which the total inhibition of visual growth, compared to the growth control well, was observed after 24 h (30). The Sensititre YeastOne YO10 (Thermo Fisher Scientific, Waltham, MA, USA) AFST was performed in accordance with the manufacturer’s recommendations. The panels were incubated at 35 ± 2°C, and the SYO color imetric MIC was recorded as the first blue well corresponding to complete growth inhibition (MIC-0) after 24 h. Alternatively, different SYO growth inhibition endpoints, defined as the lowest drug concentrations with a substantial reduction of growth (approximately 75% growth inhibition, MIC-1), a prominent reduction of growth (approximately 50% growth inhibition, MIC-2) and a slight reduction of growth (approximately 25% growth inhibition, MIC-3), relative to the drug-free control growth, were determined (Fig. 3). Each isolate was tested by both methods, using a single fungal suspension that was adjusted to the required concentration. The colony-forming unit (CFU) counts were affirmed each time via spread plate counts on Sabouraud dextrose agar plates, whereas the recommended C. krusei ATCC 6258 and C. parapsilosis ATCC 22019 strains were used as quality control strains for both methods. The CLSI and SYO MICs were evaluated via a visual inspection of the plates with the aid of a magnifying mirror by two blinded observers. In order to assess the potential variation in AMB MIC determination by SYO, a proportion of isolates (10 out of 65) were retested on different days and in different laboratories so as to determine the interday and the interlaboratory reproducibility, respectively.

### Analysis.

A head-to-head comparison of the generated MIC data sets, using the CLSI BMD as the reference methodology, was performed. For the quantitative analysis, the results of the two methods were analyzed using a paired Student’s *t* test or a Wilcoxon matched-pairs signed rank test, depending on the validity of the normality assumption. The levels of CLSI-SYO EA within ±1 and ±2 twofold dilutions were calculated. For the qualitative analysis, the CA was determined, following the CDC’s tentative AMB resistant breakpoint for C. auris (≥2 mg/L) (10). A discrepancy was considered to be a MaE when the CLSI classified an isolate as susceptible (MIC < 2 mg/L) and the SYO classified the same isolate as resistant (MIC ≥ 2 mg/L) (false resistance). A very major error (i.e., false susceptibility, classified as resistant by CLSI and as susceptible by SYO) could not be determined, as there were no CLSI AMB resistant isolates. The SYO WT-UL, which was defined as the upper MIC value at which the WT distribution ended, was determined using the ECOFFinder program (31). The WT-UL was verified in a larger collection of 205 Greek C. auris isolates that clustered in clade I and were recovered from individual patients who were hospitalized in tertiary care hospitals that were located in densely populated metropolitan areas across the country from June of 2019 to May of 2022.

## References

[1] Dennis EK, Chaturvedi S, Chaturvedi V. 2021. So many diagnostic tests, so little time: review and preview of Candida auris testing in clinical and public health laboratories. Front Microbiol 12:757835. doi:10.3389/fmicb.2021.757835. 34691009 PMC8529189

[2] Rybak JM, Barker KS, Muñoz JF, Parker JE, Ahmad S, Mokaddas E, Abdullah A, Elhagracy RS, Kelly SL, Cuomo CA, Rogers PD. 2022. In vivo emergence of high-level resistance during treatment reveals the first identified mechanism of amphotericin B resistance in Candida auris. Clin Microbiol Infect 28:838–843. doi:10.1016/j.cmi.2021.11.024. 34915074 PMC9467277

[3] Ostrowsky B, Greenko J, Adams E, Quinn M, O’Brien B, Chaturvedi V, Berkow E, Vallabhaneni S, Forsberg K, Chaturvedi S, Lutterloh E, Blog D, Bucher C, Denis RJ, Erazo R, Fernandez R, Southwick K, Zhu YC, Group C auris IW, Group C auris IW. 2020. Candida auris isolates resistant to three classes of antifungal medications — New York, 2019. MMWR Morb Mortal Wkly Rep 69:6–9. doi:10.15585/mmwr.mm6901a2. 31917780 PMC6973342

[4] Biagi MJ, Wiederhold NP, Gibas C, Wickes BL, Lozano V, Bleasdale SC, Danziger L. 2019. Development of high-level echinocandin resistance in a patient with recurrent Candida auris candidemia secondary to chronic candiduria. Open Forum Infect Dis 6. doi:10.1093/ofid/ofz262. PMC6602379 31281859

[5] Kidd SE, Crawford LC, Halliday CL. 2021. Antifungal susceptibility testing and identification. Infect Dis Clin North Am 35:313–339. doi:10.1016/j.idc.2021.03.004. 34016280

[6] Cantón E, Pemán J, Hervás D, Iñiguez C, Navarro D, Echeverría J, Martínez-Alarcón J, Fontanals D, Gomila-Sard B, Buendía B, Torroba L, Ayats J, Bratos A, Sánchez-Reus F, Fernández-Natal I, FUNGEMYCA Study Group the FS. 2012. Comparison of three statistical methods for establishing tentative wild-type population and epidemiological cutoff values for echinocandins, amphotericin B, flucytosine, and six Candida species as determined by the colorimetric Sensititre YeastOne method. J Clin Microbiol 50:3921–3926. doi:10.1128/JCM.01730-12. 23015676 PMC3503000

[7] Espinel-Ingroff A, Alvarez-Fernandez M, Cantón E, Carver PL, Chen SC-A, Eschenauer G, Getsinger DL, Gonzalez GM, Govender NP, Grancini A, Hanson KE, Kidd SE, Klinker K, Kubin CJ, Kus JV, Lockhart SR, Meletiadis J, Morris AJ, Pelaez T, Quindós G, Rodriguez-Iglesias M, Sánchez-Reus F, Shoham S, Wengenack NL, Borrell Solé N, Echeverria J, Esperalba J, Gómez-G de la Pedrosa E, García García I, Linares MJ, Marco F, Merino P, Pemán J, Pérez del Molino L, Roselló Mayans E, Rubio Calvo C, Ruiz Pérez de Pipaon M, Yagüe G, Garcia-Effron G, Guinea J, Perlin DS, Sanguinetti M, Shields R, Turnidge J. 2015. Multicenter study of epidemiological cutoff values and detection of resistance in Candida spp. to anidulafungin, caspofungin, and micafungin using the Sensititre YeastOne colorimetric method. Antimicrob Agents Chemother 59:6725–6732. doi:10.1128/AAC.01250-15. 26282428 PMC4604361

[8] Espinel-Ingroff A, Turnidge J, Alastruey-Izquierdo A, Botterel F, Canton E, Castro C, Chen YC, Chen Y, Chryssanthou E, Dannaoui E, Garcia-Effron G, Gonzalez GM, Govender NP, Guinea J, Kidd S, Lackner M, Lass-Flörl C, Linares-Sicilia MJ, López-Soria L, Magobo R, Pelaez T, Quindós G, Rodriguez-Iglesia MA, Ruiz MA, Sánchez-Reus F, Sanguinetti M, Shields R, Szweda P, Tortorano A, Wengenack NL, Bramati S, Cavanna C, DeLuca C, Gelmi M, Grancini A, Lombardi G, Meletiadis J, Negri CE, Passera M, Peman J, Prigitano A, Sala E, Tejada M. 2018. Method-dependent epidemiological cutoff values for detection of triazole resistance in Candida and Aspergillus species for the Sensititre YeastOne colorimetric broth and etest agar diffusion methods. Antimicrob Agents Chemother 63:e01651-18. 30323038 10.1128/AAC.01651-18 PMC6325216

[9] Ruiz-Gaitán AC, Cantón E, Fernández-Rivero ME, Ramírez P, Pemán J. 2019. Outbreak of Candida auris in Spain: A comparison of antifungal activity by three methods with published data. Int J Antimicrob Agents 53:541–546. doi:10.1016/j.ijantimicag.2019.02.005. 30769198

[10] Antifungal susceptibility testing and interpretation, Candida auris, fungal diseases, CDC. https://www.cdc.gov/fungal/candida-auris/c-auris-antifungal.html.

[11] Arendrup MC, Prakash A, Meletiadis J, Sharma C, Chowdhary A. 2017. Comparison of EUCAST and CLSI reference microdilution mics of eight antifungal compounds for candida auris and associated tentative epidemiological cutoff values. Antimicrob Agents Chemother 61:e00485-17. doi:10.1128/AAC.00485-17. 28416539 PMC5444165

[12] Pappas PG, Kauffman CA, Andes DR, Clancy CJ, Marr KA, Ostrosky-Zeichner L, Reboli AC, Schuster MG, Vazquez JA, Walsh TJ, Zaoutis TE, Sobel JD. 2016. Clinical practice guideline for the management of Candidiasis: 2016 update by the Infectious Diseases Society of America. Clin Infect Dis 62:e1–e50. doi:10.1093/cid/civ933. 26679628 PMC4725385

[13] Cornely OA, Bassetti M, Calandra T, Garbino J, Kullberg BJ, Lortholary O, Meersseman W, Akova M, Arendrup MC, Arikan-Akdagli S, Bille J, Castagnola E, Cuenca-Estrella M, Donnelly JP, Groll AH, Herbrecht R, Hope WW, Jensen HE, Lass-Flörl C, Petrikkos G, Richardson MD, Roilides E, Verweij PE, Viscoli C, Ullmann A J. 2012. ESCMID guideline for the diagnosis and management of Candida diseases 2012: non-neutropenic adult patients. Clin Microbiol Infect 18:19–37. doi:10.1111/1469-0691.12039. 23137135

[14] AL-Obaid I, Asadzadeh M, Ahmad S, Alobaid K, Alfouzan W, Bafna R, Emara M, Joseph L. 2022. Fatal breakthrough candidemia in an immunocompromised patient in Kuwait due to Candida auris exhibiting reduced susceptibility to echinocandins and carrying a novel mutation in hotspot-1 of FKS1. JoF 8:267. doi:10.3390/jof8030267. 35330269 PMC8953900

[15] Asadzadeh M, Mokaddas E, Ahmad S, Abdullah AA, de Groot T, Meis JF, Shetty SA. 2022. Molecular characterisation of Candida auris isolates from immunocompromised patients in a tertiary-care hospital in Kuwait reveals a novel mutation in FKS1 conferring reduced susceptibility to echinocandins. Mycoses 65:331–343. doi:10.1111/myc.13419. 34953089

[16] Chen J, Tian S, Han X, Chu Y, Wang Q, Zhou B, Shang H. 2020. Is the superbug fungus really so scary? A systematic review and meta-analysis of global epidemiology and mortality of Candida auris. BMC Infect Dis 20:827. doi:10.1186/s12879-020-05543-0. 33176724 PMC7656719

[17] CDC. 2022. Treatment and management of C. auris infections and colonization. https://www.cdc.gov/fungal/candida-auris/c-auris-treatment.html.

[18] Maphanga TG, Mpembe RS, Naicker SD, Govender NP, GERMS-SA. 2022. In vitro antifungal activity of manogepix and other antifungal agents against South African Candida auris isolates from bloodstream infections. Microbiol Spectr 10:e0171721. doi:10.1128/spectrum.01717-21. 35196811 PMC8865435

[19] Kilburn S, Innes G, Quinn M, Southwick K, Ostrowsky B, Greenko JA, Lutterloh E, Greeley R, Magleby R, Chaturvedi V, Chaturvedi S. 2022. Antifungal resistance trends of Candida auris clinical isolates, New York-New Jersey, 2016–2020. Antimicrob Agents Chemother 66:e0224221. doi:10.1128/aac.02242-21. 35007140 PMC8923207

[20] Rhodes J, Abdolrasouli A, Farrer RA, Cuomo CA, Aanensen DM, Armstrong-James D, Fisher MC, Schelenz S. 2018. Genomic epidemiology of the UK outbreak of the emerging human fungal pathogen Candida auris. Emerg Microbes Infect 7:43. 29593275 10.1038/s41426-018-0045-x PMC5874254

[21] Maphanga TG, Naicker SD, Kwenda S, Muñoz JF, van Schalkwyk E, Wadula J, Nana T, Ismail A, Coetzee J, Govind C, Mtshali PS, Mpembe RS, Govender NP, Germs-sa for. 2021. In vitro antifungal resistance of Candida auris isolates from bloodstream infections, South Africa. 10.1128/AAC.00517-21 PMC8370198 34228535

[22] CLSI. AST News update June 2022: hot topic Candida auris update: method variability with amphotericin B susceptibility testing. https://clsi.org/about/blog/ast-news-update-june-2022-hot-topic/.

[23] Clinical and Laboratory Standards Institute. 2008. Reference Method for Broth Dilution Antifungal Susceptibility Testing of Yeasts; Approved Standard—3rd Edition. CLSI document M27-A3. Wayne, PA. USA.

[24] Escandón P, Chow NA, Caceres DH, Gade L, Berkow EL, Armstrong P, Rivera S, Misas E, Duarte C, Moulton-Meissner H, Welsh RM, Parra C, Pescador LA, Villalobos N, Salcedo S, Berrio I, Varón C, Espinosa-Bode A, Lockhart SR, Jackson BR, Litvintseva AP, Beltran M, Chiller TM. 2019. Molecular epidemiology of Candida auris in Colombia reveals a highly related, countrywide colonization with regional patterns in amphotericin B resistance. Clin Infect Dis 68:15–21. 29788045 10.1093/cid/ciy411

[25] Lockhart SR, Etienne KA, Vallabhaneni S, Farooqi J, Chowdhary A, Govender NP, Colombo AL, Calvo B, Cuomo CA, Desjardins CA, Berkow EL, Castanheira M, Magobo RE, Jabeen K, Asghar RJ, Meis JF, Jackson B, Chiller T, Litvintseva AP. 2017. Simultaneous emergence of multidrug-resistant Candida auris on 3 continents confirmed by whole-genome sequencing and epidemiological analyses. CLINID 64:134–140. doi:10.1093/cid/ciw691. PMC5215215 27988485

[26] Chowdhary A, Prakash A, Sharma C, Kordalewska M, Kumar A, Sarma S, Tarai B, Singh A, Upadhyaya G, Upadhyay S, Yadav P, Singh PK, Khillan V, Sachdeva N, Perlin DS, Meis JF. 2018. A multicentre study of antifungal susceptibility patterns among 350 Candida auris isolates (2009–17) in India: role of the ERG11 and FKS1 genes in azole and echinocandin resistance. J Antimicrob Chemother 73:891–899. doi:10.1093/jac/dkx480. 29325167

[27] Ceballos-Garzon A, Garcia-Effron G, Cordoba S, Rodriguez JY, Alvarez-Moreno C, Le Pape P, Parra-Giraldo CM, Morales-López S. 2022. Head-to-head comparison of CLSI, EUCAST, Etest and VITEK®2 results for Candida auris susceptibility testing. Int J Antimicrob Agents 59:106558. doi:10.1016/j.ijantimicag.2022.106558. 35227828

[28] Kurt AF, Kuskucu MA, Balkan I, Baris A, Yazgan Z, Serife Oz A, Tosun AI, Mete B, Tabak F, Aygun G. 2021. Candida auris Fungemia and a local spread taken under control with infection control measures: First report from Turkey. Indian J Med Microbiol 39:228–230. doi:10.1016/j.ijmmb.2021.03.007. 33785243

[29] de Groot T, Puts Y, Berrio I, Chowdhary A, Meis JF. 2020. Development of Candida auris short tandem repeat typing and its application to a global collection of isolates. mBio 11:e02971-19. doi:10.1128/mBio.02971-19. 31911492 PMC6946803

[30] Clinical and Laboratory Standards Institute. 2017. Reference method for broth dilution antifungal susceptibility testing of yeasts. M27 Approved Standard-4th ed CLSI document M27-A4. Wayne, PA, USA.

[31] European Committee on Antimicrobial Susceptibility Testing. MIC and zone distributions and ECOFFs. https://www.eucast.org/mic_and_zone_distributions_and_ecoffs.

